# Erratum to “Fatty Acid Analysis and Stability of Selected Edible Oils Used in Homemade Pet Diets”

**DOI:** 10.1111/jvim.70157

**Published:** 2025-06-06

1

J. A. Larsen , J. Stockman , X. Li , and S. C. Wang , “Fatty Acid Analysis and Stability of Selected Edible Oils Used in Homemade Pet Diets,” Journal of Veterinary Internal Medicine 39, no. 3 (2025), 10.1111/jvim.70119.PMC1208698940387293

In the above‐mentioned article, conflicts of interest were omitted for Jennifer A. Larsen and Jonathan Stockman. The correct conflicts of interest should read as follows.

Jennifer Larsen is an investigator in clinical trials and other research partly or fully sponsored by Royal Canin, Nature's Variety Instinct, and Nestle Purina PetCare. She develops educational materials for Mark Morris Institute and HealthyPet Magazine, and is an advisory panel member for Purina Institute. She participated in continuing education events, as a speaker or attendee, sponsored or organized by Royal Canin, Nestle Purina PetCare, Nature's Variety Instinct, and Hill's Pet Nutrition. These professional activities for Jennifer Larsen are not relevant to the topic of the article as they do not involve edible oils, and she has no past, current, or future plans for involvement in any for‐profit entities related to this topic.

Jonathan Stockman was a paid consultant for Petco Health and Wellness Company until 2024, Konsyl Pharmaceuticals Inc. until 2023, and Mars PetCare until 2023. In addition, he has previously received a speaking honorarium from Hill‘s Pet Nutrition and research support from Hill‘s Pet Nutrition and Royal Canin, a subsidiary of Mars. He is also a paid consultant for Harrison‘s Bird Foods and was a consultant for Oxbow Animal Health until 2024. He provided learning materials and consultations for Royal Canin China, Alfie and Buddy in China, and 3dMedVision in South Korea. He participated in continuing education events and meetings sponsored or organized by Royal Canin, Nestle Purina PetCare, Nature‘s Variety Instinct, Blue Buffalo (General Mills), and Hill‘s Pet Nutrition. These professional activities for Jonathan Stockman are not relevant to the topic of the article as they do not involve edible oils, and he has no past, current, or future plans for involvement in any for‐profit entities related to this topic.

We apologize for this error.

